# Circulation of Measles Virus Genotype B3 in the Republic of the Congo Between 2023 and 2024: A Molecular Characterization Study

**DOI:** 10.1002/hsr2.71201

**Published:** 2025-08-26

**Authors:** Yanne Vanessa Thiécesse Mavoungou, Matthieu Fritz, Pembe Issamou Mayengue, Félix Koussounda Koukouikila, Igor Judicael Louzolo, Léa Gwladis Ngangoué, Léadisaelle Hosanna Lenguiya, Gabriel Ahombo, Christelle Butel, Laetitia Serrano, Martine Peeters, Eric M. Leroy, Fabien Roch Niama

**Affiliations:** ^1^ Laboratoire National de Santé Publique Brazzaville Republic of the Congo; ^2^ Faculté des Sciences et Techniques Université Marien NGOUABI Brazzaville Republic of the Congo; ^3^ TransVIHMI, Université de Montpellier, INSERM, IRD Montpellier France

**Keywords:** Central Africa, epidemiology, genotyping, measles virus, Republic of the Congo

## Abstract

**Background and Aims:**

The only previous sequence report of measles virus from the Republic of the Congo (RoC) dates back to samples collected in 2000. To update the molecular epidemiology of measles virus in the RoC, our study genotyped measles virus strains circulating in the country from January 2023 to May 2024

**Methods:**

A total of 584 serum or plasma samples were collected by the National Laboratory of Public Health through its nationwide measles surveillance activities. Among these 584 suspected cases, 231 were IgM seropositive, and 170 of them were selected for molecular detection based on their collection date.

**Results:**

Of these, 20 cases were RT‐qPCR positive, and six sequences were suitable for sequence analysis. Phylogenetic analysis confirmed that the detected measles virus belongs to genotype B3, which is one of the two genotypes currently circulating globally, alongside genotype D8. Genetic comparison revealed three closely related variants, suggesting either ongoing endemic transmission or potential importation. Notably, 45% of PCR‐positive samples had received two vaccine doses, highlighting the need for robust vaccine efficacy evaluation.

**Conclusion:**

This study emphasizes the critical role of measles virus surveillance, genotyping capacities, and genetic characterization to strengthen regional disease monitoring and containment strategies.

## 1 Introduction

The measles virus (MeV), classified under the genus *Morbillivirus* in the *Paramyxoviridae* family, is a highly infectious pathogen that can cause severe infections, especially in unvaccinated or immunocompromised individuals, as well as in children aged 0–5 years. In recent years, a global resurgence of measles has occurred worldwide, including in countries where it had previously been eliminated [1].

Nucleotide analysis between strains has enabled the identification of eight clades of the measles virus, designated A–H, and the description of 24 genotypes. Since 2021, the circulation of measles is attributed solely to the B3 and D8 genotypes [2].

In the Republic of the Congo (RoC), the measles virus has been circulating for an extended period in an endemic‐epidemic pattern [3], with several local epidemics occurring in recent years [4]. Most recently, between 2019 and 2022, the RoC experienced a steady increase in cases, with more than 537 IgM+ confirmed measles cases by 2022. Since 2022, though declining, a significant number of IgM positive cases continue to be reported.

From a genetic standpoint, data on measle viral sequences in the RoC date back to 2000, during which the circulation of the B3 genotype was identified [5]. Subsequently, no further updates have been forthcoming, representing a significant gap in the country's capacity to monitor this disease. The World Health Organization (WHO)'s strategic plan for the elimination of measles, which includes the RoC, recommends the implementation of virological surveillance of virus strains [6]. Molecular characterization of strains is a crucial tool for elucidating the dynamics of virus transmission, molecular epidemiology, and can also be used indirectly to assess vaccine strategy. The objective of this study is to update the measles sequence data in the RoC.

## 2 Methods

### 2.1 Sample Collection

Between January 2023 and May 2024, utilizing the national surveillance system established in the ROC with support from the WHO, 584 serum or plasma samples were collected from cases presenting clinical symptoms of measles infection, including generalized maculopapular rash and fever. Information on age, sex, vaccination status (as documented in the vaccination records), sample collection date, the date of onset of the rash, city of residence, and department were also recorded. Other of the following symptoms: cough, coryza (runny nose), or conjunctivitis (red eyes) are also reported if presented.

### 2.2 Ethical Approval and Consent to Participate

The samples were obtained through the measles surveillance system. As this infection is notifiable, an ethical agreement was not needed. However, a statement summarizing the objectives of the surveillance was read to each parent in French or in one of the two national languages (Lingala and Kituba). The interviews were conducted in private to guarantee the confidentiality of the information collected, in accordance with the Declaration of Helsinki (WMA ‐ World Medical Association).

### 2.3 Laboratory Analysis

Serum were tested by IgM detection using ELISA (IgM kit, EUROIMMUN Lübeck, Germany) at the National Public Health Laboratory of Brazzaville (LNSP) as previously describe [4].

Viral RNA was extracted from serum or plasma using the Qiamp viral RNA kit (Qiagen). The presence of MeV RNA in samples was assessed using an optimized protocol with primers MVTaqfw (nt 584–607), MVTaqrv (nt 697–675), and MVTaq probe (nt 634–657), which target the nucleoprotein gene as previously described [7]. Amplification was performed with the SuperScript III Platinum One‐Step RT‐qPCR System (Thermo Fisher Scientific).

### 2.4 Genotyping

RNA from RT‐qPCR‐positive patients was then sequenced in UMR TransVIHMI, Montpellier, France. These RNA samples were also analyzed by MeV genotyping RT‐PCR using the MeV216 and MeV214 primers, which target a 634 bp fragment of the N‐gene [8]. The PCR products were sequenced using the Sanger method, and the resulting sequences were edited with DNAStar software (Version: 17.5.0).

Phylogenetic analysis was performed on 450 nucleotides of the N‐gene in accordance with WHO recommendations for measles virus strain genotyping. Sequences were aligned using the Clustal Omega algorithm with the 29 reference sequences representing all known genotypes, as well as the two most recent sequences from the Republic of the Congo sampled in 2000, closely related sequences for each identified variant through BLAST, and recent measles sequences from Cameroon and the Central African Republic [9, 10]. IQ‐TREE v1.6.12 [11] was used to generate a maximum likelihood tree, applying the best‐fit nucleotide substitution model for our data set. Branch support was estimated using the UltraFast bootstrap method with 1000 replicates, ensuring the reliability of the phylogenetic analyses at each branch node [12]. Phylogenetic trees were formatted and annotated using iTOL v6 [13]. All sequences obtained in this study were named according to the WHO recommendations and submitted to GenBank under accession number **PQ409325** to **PQ409330**.

## 3 Results

Among the 584 suspected cases, 231 were confirmed positive by IgM detection (IgM + ). From the 231 IgM‐positive cases, 170 samples were selected for RNA detection based on their collection date, ensuring they were within 10 days of symptom onset to align with the potential viremia window.

**Figure 1:**
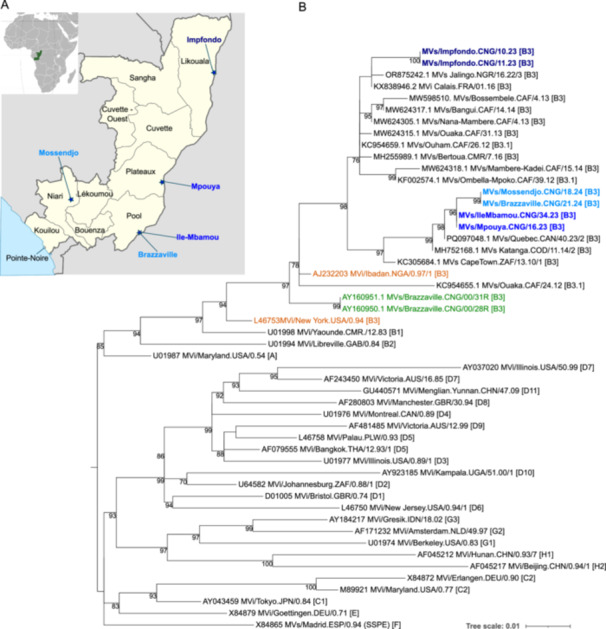
Location and phylogenetic tree using the C‐terminal 450 nucleotides coding for the N gene sequence. (A) Locations (blue star) of studied sequences are indicated on the map. (B) Sequences from this study are marked in bold blue and fall within genotype B3. Each variant is represented by a distinct shade of blue, with locations on the map indicated by the corresponding color. The 2000 sequences from the Republic of the Congo are shown in green. WHO reference sequences for genotype B3 are in orange, while WHO reference sequences for other genotypes are also included. Other genotype B3 sequences are indicated. The genotype of each sequence is indicated in square brackets at the end of their name. Bootstrap values (> 70%) are shown on the nodes.

Overall, 20 of 170 IgM+ samples (11.8%) tested RT‐qPCR positive with CT values ranging from 31.17 to 37.23. Among these 20 patients, 9 were males (45%), 11 were females (55%). The patients' ages ranged from 1 to 18 years, with a median age of 4 years. These 20 patients came from 7 of the 12 departments of the RoC, spanning from the north‐east (Impfondo), near the border with the Democratic Republic of the Congo (DRC), to the south‐west (Pointe‐Noire) (Figure 1A). Among these 20 PCR+ patients, 4 (20%) were unvaccinated, 7 (35%) had received one dose and 9 (45%) had received two doses. Among the 20 PCR+ samples, 6 (30%) were positive in the genotyping assay in the N gene and suitable for sequence and phylogenetic analysis. The CT values for these samples ranged between 31.17 and 33.1 (Table 1).

**Table 1:** Characteristics of MeV confirmed IgM patients with RT‐qPCR positive result collected in Republic of the Congo from January 2023 to May 2024.

Id Patient	Genbank	WHO nomenclature	City	Department	Age (Years)	Sex	Date of onset of the rash	Date of sampling	Vaccination status	CT value
23‐063	PQ409327	MVs/Impfondo.CNG/10.23[B3]	Impfondo	Likouala	5	M	11/03/2023	13/03/2023	2	33.23
23‐067	PQ409329	MVs/Impfondo.CNG/11.23[B3]	Impfondo	Likouala	9	F	14/03/2023	15/03/2023	2	32.85
23‐123	PQ409326	MVs/Mpouya.CNG/16.23[B3]	Mpouya	Plateaux	7	F	21/04/2023	26/04/2023	1	32.47
23‐286	PQ409325	MVs/IleMbamou.CNG/34.23[B3]	Ile‐Mbamou	Brazzaville	1	M	21/08/2023	27/08/2023	2	31.17
24‐161	PQ409328	MVs/Mossendjo.CNG/18.24[B3]	Mossendjo	Niari	18	F	01/05/2024	09/05/2024	1	33.1
24‐181	PQ409330	MVs/Brazzaville.CNG/21.24[B3]	Brazzaville	Brazzaville	4	F	26/05/2024	29/05/2024	0	31.47
23‐037	N/A	N/A	Oyo	Cuvette	3	F	23/02/2023	01/03/2023	2	35.25
23‐066	N/A	N/A	Impfondo	Likouala	3	F	14/03/2023	14/03/2023	2	32.54
23‐083	N/A	N/A	Brazzaville	Brazzaville	1	M	19/03/2023	25/03/2023	1	34.92
23‐152	N/A	N/A	Brazzaville	Brazzaville	8	F	07/05/2023	09/05/2023	1	36.54
23‐214	N/A	N/A	Mossaka	Cuvette	5	M	21/06/2023	23/06/2023	2	35.13
23‐215	N/A	N/A	Mossaka	Cuvette	4	M	19/06/2023	23/06/2023	2	36.49
23‐277	N/A	N/A	Brazzaville	Brazzaville	5	F	15/08/2023	16/08/2023	2	32.78
23‐386	N/A	N/A	Pointe‐Noire	Pointe‐Noire	8	M	30/11/2023	03/12/2023	1	37.14
24‐005	N/A	N/A	Brazzaville	Brazzaville	1	M	06/01/2024	16/01/2024	0	31.27
24‐030	N/A	N/A	Brazzaville	Brazzaville	2	F	13/02/2024	15/02/2024	0	33.19
24‐060	N/A	N/A	Impfondo	Likouala	3	F	29/02/2024	05/03/2024	0	34.08
24‐061	N/A	N/A	Impfondo	Likouala	5	M	02/03/2024	05/03/2024	2	37.12
24‐126	N/A	N/A	Mpouya	Plateaux	2	F	24/04/2024	25/04/2024	1	36.96
24‐127	N/A	N/A	Mpouya	Plateaux	2	M	25/04/2024	25/04/2024	1	37.23

Abbreviations: CT, cycle threshold; F, female; Id, identification; M, male; N/A, not applicable; WHO, World Health Organization.

Analysis of these six sequences revealed that two sequences (from patients 23‐063 and 23‐067), both from Impfondo, and with similar dates of rash onset, were identical. The same was observed for the sequences of patients 23‐286 and 23‐123 (Ile‐Mbamou and Mpouya), as well as for patients 24‐161 and 24‐181 (Mossendjo and Brazzaville) (Figure 1, Table 1). The sequence from Impfondo shares 96.67% and 96.22% nucleotide identity with the sequences from Mpouya and Ile‐Mbamou, and from Brazzaville and Mossendjo, respectively. In contrast, the sequences from Mpouya and Ile‐Mbamou show 99.5% identity with those from Mossendjo and Brazzaville. Comparison of these six sequences with the WHO reference sequences for measles showed that all sequences belonged to genotype B3 (Figures 1 and S1).

## 4 Discussion

The aim of this study was to characterize the genotype(s) of the measles virus circulating in the RoC between January 2023 and May 2024. The epidemiological data from RT‐PCR positive patients align with those observed in a larger epidemiological study in the RoC, which analyzed 537 IgM+ confirmed cases over a 4‐year period (2019–2022) [4]. To our knowledge, this is the first study of its kind since the RoC joined the Strategic Plan for Measles Elimination in Africa in 2012. Our study showed an RT‐PCR positivity rate of 11.7% in these samples, which is lower than the rates reported in previous studies [9, 14, 15]. This difference may be explained by suboptimal transport and storage conditions that could have affected the integrity of the samples. The first and only sequences available for the RoC dates back to samples obtained in 2000, during which the B3 genotype was identified [5]. Our study shows that genotype B3 remains one of the circulating genotypes in the Republic of the Congo. However, our data do not fully represent the diversity of genotypes or variants circulating in the country, due to the limited number of samples analyzed. A larger study is therefore required to address this limitation. However, this finding aligns with global data on measles virus circulation, particularly in Africa, where genotype B3 is predominantly reported and responsible for recent epidemics, while genotype D8 is more commonly detected in Europe, the Americas, and Asia [2, 9, 16].

A significant number (9 out of 20) of RT‐PCR positive patients had received two vaccine doses, which is consistent with the high proportion of double‐dose positive cases reported in a previous study in the RoC [17]. The recommended vaccination schedule in the RoC includes two doses, administered at 9 and 15 months of age. Our study was unable to assess IgG antibody avidity. Consequently, we could not conclusively determine whether these cases were due to primary vaccine failure or secondary vaccine failure. Although vaccination dates were unavailable, based on age, one patient (23‐286) may have been experiencing postvaccination symptoms. However, this study did not detect the Edmonston vaccine strain in this patient or any others, indicating infection by a circulating strain and thereby confirming vaccine failure. Given the genetic proximity of the sequences reported in this study to the Edmonston vaccine strain, vaccine failure is unlikely to be due to viral escape. Instead, it may be linked to issues with the cold chain or exposure of vaccines to light, as the measles vaccine is highly photosensitive. Furthermore, inadequate training of personnel in vaccine storage and handling could exacerbate these challenges.

These findings indicate the presence of three different variants across various areas and sampling dates. Due to the limited data available, including epidemiological information, the small number of sequences, and the fact that only the N450 region could be sequenced, it is not possible to distinguish between ongoing endemic transmission and multiple importation events. The Republic of the Congo and the Democratic Republic of the Congo are neighbouring countries with a high level of human exchange. Thus, potential importations may have originated from the DRC, which has experienced the largest measles epidemic in recent years [18] and continues to face high virus circulation in bordering provinces such as Equateur and Maindombe.

As part of the global effort for measles eradication, it is necessary to establish virological surveillance, including the genetic characterization of measles strains in the RoC [19]. Above all, it is crucial to monitor vaccine efficacy in light of potential strain changes and the possible emergence of variants. While the N450 region is sufficient for genotyping, recent studies have demonstrated that larger part of the genome or whole‐genome sequencing is necessary for a better understanding of epidemiological investigations [6]. It is therefore necessary to improve the time and conditions under which samples are transported, implement oropharyngeal sampling during epidemics, and train staff in appropriate sampling techniques to enhance the sensitivity of molecular detection.

## Author Contributions

**Yanne Vanessa Thiécesse Mavoungou:** conceptualization, data curation, formal analysis, investigation, methodology, resources, validation, visualization, writing – original draft, writing – review and editing. **Matthieu Fritz:** conceptualization, data curation, formal analysis, investigation, methodology, validation, visualization, writing – original draft, writing–review and editing. **Pembe Issamou Mayengue:** conceptualization, data curation, validation, writing – review and editing. **Félix Koussounda Koukouikila:** data curation, methodology, validation, writing – review and editing. **Igor Judicael Louzolo:** conceptualization, data curation, formal analysis, investigation, methodology, validation, writing – review and editing. **Léa Gwladis Ngangoué:** conceptualization, data curation, methodology, resources, validation, writing – review and editing. **Léadisaelle Hosanna Lenguiya:** conceptualization, data curation, methodology, validation, writing – review and editing. **Gabriel Ahombo:** conceptualization, data curation, validation, writing – review and editing. **Christelle Butel:** data curation, formal analysis, methodology, validation, writing – review and editing. **Laetitia Serrano:** data curation, formal analysis, methodology, validation, writing – review and editing. **Martine Peeters:** conceptualization, funding acquisition, resources, writing – review and editing. **Eric M. Leroy:** conceptualization, data curation, funding acquisition, project administration, validation, writing – review and editing. **Fabien Roch Niama:** conceptualization, data curation, funding acquisition, project administration, resources, supervision, validation, writing – review and editing.

## Conflicts of Interest

The authors declare no conflicts of interest.

## Transparency Statement

The lead author Eric M. Leroy, Fabien Roch Niama affirms that this manuscript is an honest, accurate, and transparent account of the study being reported; that no important aspects of the study have been omitted; and that any discrepancies from the study as planned (and, if relevant, registered) have been explained.

## Supporting information


**Supporting Figure S1:** Phylogenetic tree of sequences from this study alongside reference sequences. Sequences from this study are marked in bold blue and fall within genotype B3. Each variant is represented by a distinct shade of blue. WHO reference sequences for genotype B3 are in orange, while WHO reference sequences for other genotypes are also included. The genotype of each sequence is indicated in square brackets at the end of their name. Bootstrap values (> 70%) are shown on the nodes.

Supporting information

## Data Availability

The data that support the findings of this study are available from the corresponding author upon reasonable request.
