# Supplementary material for: Circulation of Measles Virus Genotype B3 in the Republic of the Congo Between 2023 and 2024: A Molecular Characterization Study
Source: Health Sci Rep. 2025 Aug 26;8(9):e71201. doi: 10.1002/hsr2.71201 (PMC12379835; doi:10.1002/hsr2.71201)
Supplement: Supplementary file 2 — Supporting information [file HSR2-8-e71201-s001.docx]

Figure S1 Phylogenetic tree of sequences from this study alongside reference sequences. Sequences from this study are marked in bold blue and fall within genotype B3. Each variant is represented by a distinct shade of blue. WHO reference sequences for genotype B3 are in orange, while WHO reference sequences for other genotypes are also included. The genotype of each sequence is indicated in square brackets at the end of their name. Bootstrap values (>70%) are shown on the nodes.
